# Mathematical Modeling of Initial Exothermic Behavior and Thixotropic Properties in Nanoclay-Enhanced Cementitious Materials

**DOI:** 10.3390/ma17071502

**Published:** 2024-03-26

**Authors:** Peng Xiao, Xi Chen, Donglin Cao, Yong Yuan, Ying Dai, Neven Ukrainczyk, Eddie Koenders

**Affiliations:** 1 Institute of Construction and Building Materials, Technical University of Darmstadt, Franziska-Braun-Str. 3, 64287 Darmstadt, Germany; xiao@wib.tu-darmstadt.de (P.X.); cao@wib.tu-darmstadt.de (D.C.); koenders@wib.tu-darmstadt.de (E.K.); 2 College of Civil Engineering and Architecture, Jiaxing University, Jiaxing 314001, China; 123cx@alumni.tongji.edu.cn; 3 College of Civil Engineering, Tongji University, Shanghai 200092, China; yuany@tongji.edu.cn; 4 School of Aerospace Engineering and Applied Mechanics, Tongji University, Zhangwu Road 100, Shanghai 200092, China; ydai@tongji.edu.cn

**Keywords:** cementitious materials, additive manufacturing, nanoclay, cement hydration, nucleation, initial exothermic reactions, thixotropy, mathematical modeling

## Abstract

In the realm of cementitious materials, integrating nanoclay shows promise in enhancing properties relevant to additive manufacturing. This paper presents a novel mathematical model that combines simple empirical dissolution/nucleation Avrami-like kinetics with a thixotropic kinetics equation. To analyze the initial exothermic peak, two sets of the calculation parameter function are built to describe the exothermic rate as a function of time, following an exponential pattern. This allows for the prediction of the changes in cumulative heat and heat rate during hydration, considering different concentrations of nanoclay. In the rheological aspect, the relationship between shear stress, shear rate, and time is modeled as a combination of exponential dependencies. This enables the prediction of the variations in shear stress with one variable while holding the other constant (either time or shear rate). By integrating these aspects, this model effectively describes both the first exothermal peak and the rheological behavior during cement hydration with the inclusion of nanoclay. Validated against experimental results, these models demonstrate good accuracy (overall below 3% error), reliability, and applicability. The findings offer valuable insights into the thermal and rheological aspects of concrete printing, enabling informed design decisions for both scientific and industrial applications.

## 1. Introduction

Concrete printing technology is an emerging construction technology offering various benefits, including design flexibility, material savings, faster construction times, reduced costs, and improved safety [1,2]. The success of three-dimensional printing of cementitious materials or concrete (3DPC), also known as additive manufacturing, largely depends on the viscoelastic properties and thermal behavior of the materials [3,4].

One major challenge in concrete printing is ensuring that 3DPC in its fresh state has the right balance of fluidity and stiffness. This balance is crucial for extrudability and to prevent deformation [5,6]. These requirements are significantly influenced by the rheological properties and can also be gauged by heat release behavior [7]. The rheological properties of fresh mortar are influenced by various factors, including the concrete’s age, shear history, and temperature [8]. Understanding and regulating these factors is vital to ensure the quality and stability of the final product, especially during the placement at the job site [9]. An interdisciplinary approach is essential for investigating and describing the fresh rheology of cementitious pastes, employing analytical techniques, microstructural modeling, and experimental rheometry [10].

Among different types of nanoparticles (NPs) in the mixture of 3DPC, particularly, the addition of nanoclay (NC) particles has been shown to enhance the shape stability of semi-flowable self-consolidating concrete by improving the thixotropy of the concrete [11]. The thixotropic properties of cement mortar can be improved through several mechanisms: the surfaces and edges of most clay particles being charged [12]; irregular microstructures can effectively build up a strong network; nanoclay particles as nano-fillers can fill the gaps between cement particles, resulting in an interlocking microstructure [13]. These effects are also reflected in the early exothermic behavior, e.g., an increase in the rate of heat generation [14]. According to the exothermic characteristics of hydration, five stages were divided, including the rapid reaction period, induction period, acceleration period, deceleration period, and recession period [15]. The first peak of heat flow that occurs during the rapid reaction period is primarily due to the silicate reaction, which involves the dissolution of alite and the precipitation of portlandite and calcium silicate hydrate (CSH) gel [16]. During the first few minutes to an hour after mixing, rapid heat generation occurs, which may lead to issues during the printing process. Furthermore, an increase in temperature rise can lead to early-age cracking and delayed expansion of ettringite in large-volume concrete structures [17]. 

Due to the combined and inter-influenced effects of these mechanisms, accurately describing the early exothermic behavior and flow characteristics of cements incorporating NPs, especially nanoclay, can be challenging [18]. Some scholars have dedicated their efforts to investigating the impact of various factors on the properties of 3DPC with NPs or nanoclay, such as Kozior and Kundera [19], who analyzed the viscoelastic properties of cellular models created with PolyJet Matrix technology, using Mathematica and original data for statistical analysis to approximate relaxation curves and identify optimal rheological parameters for future material and geometry matching in 3DPC components. Ayegba [20] evaluated the energy efficiency, carbon emissions, and thermal comfort of air cavity 3DPC building envelopes compared to insulated ones, using both experimental results and numerical optimization analysis to find combinations with optimal energy, carbon, and thermal performance for four different mixes. Nodehi [21] critically examined the durability of 3DPC, focusing on how printing parameters, mixture compositions, and key materials influence its shrinkage, porosity, freeze-thaw, fire, chemical, and acid resistance, and offered suggestions for enhancing durability in various environments. Han [22] developed a microscale-based numerical model for studying the coupled hygro-thermo-mechanical behavior of the 3DPC at elevated temperatures. Şahin and Mardani [23] addressed the primary challenge in 3DPC of weak interlayer-bonding strength (IBS), discussing its influencing factors like physical, material, and printer-induced effects.

The models reported so far predominantly employ a combination of experimental-normalized models or numerical simulation methods. The former’s universality is limited by experimental constraints, while the latter requires a foundational understanding of specific software and simulation methodologies, along with the possibility of certain intermediary parameters being ambiguously defined. Mathematical models are often derived from existing mature models, giving them a more profound physical significance. Through the language of mathematical modeling, various factors influencing the target properties of the study can be conveniently parameterized, making these models widely applicable across different fields. Developing mathematical models that align with experimental results can provide valuable insights for the quantitative characterization, quality assessment, and design of experimental and industrial methods [24]. Wallevik [9] extended the Hattori–Izumi theory [25] using a semi-microstructural approach, introducing three yield stress variables related to permanent, reversible, and chemically formed breakable linkages. The recently adapted statistical model named BreakPro [26], integrates aspects from the YODEL model [27,28], Kapur’s model [29], and includes interparticle bonds breaking probability, encompassing orientation effects, boundary nucleation, growth effects, and interparticle forces. However, successful mathematical models like these do not intricately consider the influence of NPs on cementitious materials. In Ridi, Francesca’s study [30], various calorimetric methods were employed to monitor the hydration reaction of pure tricalcium silicate and the resulting hydration curves were modeled using the Boundary Nucleation and Growth Model to extract thermodynamic parameters for the early stages of the reaction. Bai, Shuai [31] investigated the impact of different nano-silica dosages and hydration temperatures on early-age cement hydration heat by isothermal calorimetry and employed a hydration kinetics model to analyze hydrate nucleation and growth rates. Although these works primarily focused on the hydration processes spanning several hours to tens of hours, the theories utilized, including by Avrami [32] and Cahn [33], can also serve as the foundation for our research.

In this paper, we aim to find a suitable mathematical model that can describe the early exothermic behavior and thixotropic properties of cement after the addition of nanoclay, considering various factors that influence these properties. Due to the intricate nature of cement dissolution, which intertwines with nucleation mechanisms, especially in the early stages, this paper employs a simplified empirical model to firstly describe the early-stage exothermic behavior, where a particle nucleation part was derived based on the Avrami [32], and Cahn [33,34] theories. Secondly, a rheological part in this model which represents the thixotropic properties in the early stage was further derived based on the work from Cheng and Evans [35]. Then we presented and compared experimental test results with this mathematical model to better understand the effect of nanoclay particles on the early hydration process and the flow characteristics. The utilization of such a model is justified by the subsequent observation of significantly increased dissolution/nucleation rates associated with increasing nanoclay additions, providing a compelling argument for its implementation. 

## 2. Mathematical Model Establishment

### 2.1. The First Exothermic Peak

Initially, based on the existing research outcomes, we integrated the classical Avrami and Cahn equations along with their expanded forms into our research context—cement-based materials—the process is outlined as follows:

In the exothermic process of chemical reactions, it is often considered that the degree of reaction is positively correlated with heat release, overall, the hydration degree $\alpha_{d}$ can be expressed from heat release as [36]:

(1) $$\alpha_{d}=\frac{Q\left(t\right)}{Q_{\max}}$$

where $\alpha_{d}$ is the degree of hydration, *t* is time, and $Q\left(t\right)$ is the heat release function (heat of dissolution) of time *t*, $Q_{\max}$ is the maximum heat release. The duration of the first exothermic peak of hydration is short (usually within 60 min after the end of mixing), and there are many influencing factors (temperature, C3S, C3A, admixtures, dopants, etc.). In general, the heat flow model for the first exothermic peak could be defined as a function of temperature *T* and *t* as [36]:

(2) $$Q_{\max}\frac{d\alpha_{d}}{dt}=F\left(T,t\right)$$

Since $\alpha_{d}$ is the degree of hydration, in the context of this research background, it can be considered that $Q_{\max}\frac{d\alpha_{d}}{dt}$ is regarded as a heat flow. When *T* = *T*_0_, we can get a condition function as Equations (3) and (4):

(3) $$F\left(T_{0},t\right)=f\left(t\right)$$

(4) $$Q_{\max}\frac{d\alpha_{d}}{dt}=f\left(t\right)$$

In the whole hydration process, the main chemical reactions involved are shown as [37]:

(5) $$2\left(3\mathrm{CaO}\cdot {\mathrm{SiO}}_{2}\right)+6H_{2}O=3\mathrm{CaO}\cdot 2{\mathrm{SiO}}_{2}\cdot 3H_{2}O+3\mathrm{Ca}{\left(\mathrm{OH}\right)}_{2}\;2\left(2\mathrm{CaO}\cdot {\mathrm{SiO}}_{2}\right)+4H_{2}O=3\mathrm{CaO}\cdot 2{\mathrm{SiO}}_{2}\cdot 3H_{2}O+\mathrm{Ca}{\left(\mathrm{OH}\right)}_{2}\;3\mathrm{CaO}\cdot {\mathrm{Al}}_{2}O_{3}+6H_{2}O=3\mathrm{CaO}\cdot {\mathrm{Al}}_{2}O_{3}\cdot 6H_{2}O\;4\mathrm{CaO}\cdot {\mathrm{Al}}_{2}O_{3}\cdot {\mathrm{Fe}}_{2}O_{3}+7H_{2}O=3\mathrm{CaO}\cdot {\mathrm{Al}}_{2}O_{3}\cdot 6H_{2}O+\mathrm{CaO}\cdot {\mathrm{Fe}}_{2}O_{3}\cdot H_{2}O$$

During the first hour of cement hydration, mainly ettringite and CSH would be considered, while Ca(OH)_2_ forms just before setting and ferrite phases are assumed to be not relevant because of the too slow reaction rate [38,39]. Regardless of the complexity of involved reactions, if the density of cement particles and the heat release per unit mass remain constant, the initial hydration process can be described as:

(6) $$\frac{dX}{dt}=\frac{d\alpha_{d}}{dt}$$

where X is the volume fraction consumed by the chemical reaction. Referring to Avrami [32] and Cahn [33] theories, the nucleation growth model of the particles is considered to exhibit an exponential relationship with time:

(7) $$\frac{d\alpha_{d}}{dt}=K_{1}\exp\left(\eta t^{n}\right)$$

$K_{1}$, η, and n are calculating variables, according to Avrami’s crystalline model of nuclear growth, the variables are expressed as the form in:

(8) $$K_{1}=nk_{\mathrm{avr}}^{n}t^{n-1},\;\eta =-k_{\mathrm{avr}}^{n}$$

According to the Cahn model, the variables are expressed as follows:

(9) $$K_{1}=\pi D^{2}I_{s}\left(1+K\right),\;\eta =-\pi D^{2}I_{s},\;n=1$$

where *K*_avr_, *n*, *D*, and *I*_s_ are all constant parameters referring to material properties. Through mathematical derivation and simplification by us, all unknown parameters are ultimately transformed into equations dependent on time and nanoclay content. This process yields the relationship between heat release and both time and nanoclay content. The specific derivation process is as follows:

Based on our research objectives, the following Equations (10)–(12) have been formulated by us, wherein $K_{1}$ is considered as a time-dependent function:

(10) $$K_{1}=F\left(t\right)$$

when *t* > 0, a function *H*(*t*) is constructed, thereby establishing a time-dependent equation describing the relationship between heat release and time:

(11) $$F\left(t\right)=\frac{1}{Q_{\max}}H\left(t^{\lambda }\right)t^{0.5}$$

where λ is a real number to be determined. Expanding *H* on $t^{\lambda }$, the following can be derived:

(12) $$H\left(t^{\lambda }\right)\approx t^{0.5}\sum_{i=0}^{n_{1}}{\left(V_{i}t^{\lambda }\right)}^{i}$$

where *V_i_* (*i* = 1, 2, 3, …, *n*_1_) are the calculating parameters. For simplifying treatment, let *n* = 1 [33] in Equation (7) and substituting Equations (10)–(12) into Equation (7), the following form could be deduced [40]:

(13) $$Q_{\max}\frac{d\alpha_{d}}{dt}=t^{0.5}\sum_{i=0}^{n_{1}}{\left(V_{i}t^{\lambda }\right)}^{i}\exp\left(\eta t\right)$$

Taking the 1st order approximation, when temperature is constant, there is a four-parameter model as

(14) $$Q_{\max}\frac{d\alpha_{d}}{dt}\approx \left(V_{0}t^{0.5}+V_{1}t^{b}\right)\exp\left(\eta t\right)$$

when *n* = 1, it is directly reduced to the Cahn model of crystal nucleus growth. 

In Equation (14), $V_{0}$, $V_{1}$, *b*, and η are calculating parameters, they could be calibrated by thermal material factors such as cement material components, admixtures, and the usage of additives from experimental measured values. If we further confine the cement material components and the measured temperature, the model will only be influenced by the admixture, when the amount of admixture (such as silica fume or blast furnace slag) remains unchanged, only the influence of nanoclay needs to be considered. To describe the influence of nanocaly, an influence parameter function was introduced to the above four-parameter model. After establishing the basic definitions above, we can introduce a nanoclay influence factor function to further derive Equation (14) as follows:

(15) $$Q_{\max}\frac{d\alpha_{d}}{dt}\approx K_{2}\left(V_{0}t^{0.5}+V_{1}t^{b}\right)\exp\left(\eta t\right)$$

where *K*_2_ is the nanoclay influence factor function, which is the function of nanoclay content and time:

(16) $$K_{2}=K_{2}\left(n_{\mathrm{NC}},t\right)$$

where $n_{\mathrm{NC}}$ is the content of nanoclay. Using mathmatical tool to expand *K*_2_ shows:

(17) $$K_{2}=K_{20}+\sum_{i=0}^{+\infty }f_{i}\left(n_{\mathrm{NC}}\right){\left[h\left(t\right)\right]}^{i}$$

where $K_{20}$ is the value $K_{2}$ at a known time and nanoclay content, $h\left(t\right)$ is a function of *t* in the general expression form. When $h\left(t\right)=t$, the above equation is the binary Taylor expansion for $K_{2}$. For $f_{i}\left(n_{\mathrm{NC}}\right)$, based on the measured results, and considering the oscillatory nature of $n_{\mathrm{NC}}$, it is always possible to use the trigonometric function and the power series at $n_{\mathrm{NC}}=0$ to get the joint approximation as:

(18) $$f_{i}\left(n_{\mathrm{NC}}\right)=\sum_{j=1}^{+\infty }W_{j}\sin\left(\beta_{j}n_{\mathrm{NC}}\right)+\sum_{j=1}^{+\infty }U_{j}{\left({n_{\mathrm{NC}}}^{\zeta }\right)}^{j}$$

For the $h\left(t\right)$, it can be expanded as

(19) $$h\left(t\right)=\sum_{j=0}^{+\infty }CC_{j}{\left(t^{\xi }\right)}^{j}$$

where $W_{1}$, $\beta_{1}$, $U_{1}$, ζ, ξ, and $CC_{j}$ are the calculating parameters, and to prevent singularities at ξ < 0, the expansion of $h\left(t\right)$ will be:

(20) $$h\left(t\right)=\sum_{j=0}^{+\infty }CC_{j}{\left(1+t^{\xi }\right)}^{j}$$

Taking the above Equations (18) and (20) into the 1st order approximation of Equation (17) for simplicity of solution, we can get:

(21) $$K_{2}\approx K_{20}+\left(W_{1}\sin\left(\beta_{1}n_{\mathrm{NC}}\right)+U_{1}{n_{\mathrm{NC}}}^{\zeta }\right)\left(1+t^{\xi }\right)$$

When $n_{\mathrm{NC}}=0$, Equation (21) is reduced to the net pulp expression, and

(22) $$K_{20}=1$$

When considering the effect of nanoclay, Equations (21) and (22) are substituted into Equation (15) to get the first exothermic peak heat flow model as:

(23) $$Q_{\max}\frac{d\alpha_{d}}{dt}\approx \left[1+\left(W_{1}\sin\left(\beta_{1}n_{\mathrm{NC}}\right)+U_{1}{n_{\mathrm{NC}}}^{\zeta }\right)\left(1+t^{\xi }\right)\right]\left(V_{0}t^{0.5}+V_{1}t^{b}\right)\exp\left(\eta t\right)$$

where, $V_{0}$, $V_{1}$, *b*, and η are the known parameters of the first exothermic peak for a given net slurry. In addition, $W_{1}$, $\beta_{1}$, $U_{1}$, ζ, and ξ are served as calculating parameters, to simplify the impact brought by factors other than NC and time in different systems (such as NC from different sources or different mixing processes). Calculating parameters need to be calibrated for different research systems. For the same research system, they only need to be calibrated once. Calibration data can be arbitrarily selected from known experimental data, as long as they belong to the same research system.

### 2.2. The Rheological Behavior

Similar to determining the equation for the first exothermic peak in cement hydration reactions, we initially build upon existing research findings to establish a foundational equation tailored specifically for the study of cement-based materials: 

Based on the research of Cheng and Evans [35], considering the thixotropic effects, the rheological basic model of the slurry is formulated as follows:

(24) $$\tau =\eta \left(\lambda ,\dot{\gamma }\right)\dot{\gamma }$$

where λ is the structural parameter, Papo expanded its function as [41]:

(25) $$\tau =f_{0}\left(\dot{\gamma }\right)+f_{1}\left(\dot{\gamma }\right)\lambda +\cdots +f_{n}\left(\dot{\gamma }\right)\lambda^{n}$$

When employing mathematical methods to derive models, retaining only the most significant influencing parameters or functions can simplify the calculation process. If only considering to the 1st order, then Equation (25) can be reduced to:

(26) $$\tau \approx f_{0}\left(\dot{\gamma }\right)+f_{1}\left(\dot{\gamma }\right)\lambda$$

In Papo’s expanding process, the controlling differential equation with respect to λ is given by:

(27) $$\frac{d\lambda }{dt}=K_{1}\left(\dot{\gamma }\right){\left(1-\lambda \right)}^{p}-K_{2}\left(\dot{\gamma }\right)\lambda^{q}$$

where $K_{1}\left(\dot{\gamma }\right)$ and $K_{2}\left(\dot{\gamma }\right)$ are the rates that cause coalescence (build-up) and decomposition (breakdown) of cement particles in thixotropy, *p* and *q* are the coalescence and decomposition orders, and according to Zhang et al. [42], the cement paste can be considered as a 1st order equation as:

(28) $$\frac{d\lambda }{dt}=K_{1}\left(\dot{\gamma }\right)\left(1-\lambda \right)-K_{2}\left(\dot{\gamma }\right)\lambda$$

When the shear rate is constant, the function of λ can be reconstructed by us as follows:

(29) $$\lambda =\frac{K_{1}\left(\dot{\gamma }\right)}{K_{1}\left(\dot{\gamma }\right)+K_{2}\left(\dot{\gamma }\right)}+(\lambda_{t=t_{0}}-\frac{K_{1}\left(\dot{\gamma }\right)}{K_{1}\left(\dot{\gamma }\right)+K_{2}\left(\dot{\gamma }\right)})e^{-\left(K_{1}\left(\dot{\gamma }\right)+K_{2}\left(\dot{\gamma }\right)\right)\left(t-t_{0}\right)}$$

where $\lambda_{t=t_{0}}$ is the initial value of λ at the resting time *t* = *t*_0_. 

Substituting Equation (29) into (26), we can get:

(30) $$\begin{array}{cc} \tau \approx f_{0}\left(\dot{\gamma }\right)+f_{1}\left(\dot{\gamma }\right) & \frac{K_{1}\left(\dot{\gamma }\right)}{K_{1}\left(\dot{\gamma }\right)+K_{2}\left(\dot{\gamma }\right)}\\ & +f_{1}\left(\dot{\gamma }\right)(\lambda_{t=t_{0}}-\frac{K_{1}\left(\dot{\gamma }\right)}{K_{1}\left(\dot{\gamma }\right)+K_{2}\left(\dot{\gamma }\right)})e^{-\left(K_{1}\left(\dot{\gamma }\right)+K_{2}\left(\dot{\gamma }\right)\right)\left(t-t_{0}\right)} \end{array}$$

When thixotropy is not considered, $f_{0}\left(\dot{\gamma }\right)$ is a conventional rheological instantiation model. The Herschel–Bulkley model is in better agreement with the measured rheological value [41], and its calibration is used in this article as:

(31) $$f_{0}\left(\dot{\gamma }\right)\approx C_{0}+C_{1}{\dot{\gamma }}^{D}$$

where *C*_0_, *C*_1_, and *D* are all calculating parameters. According to the results of Vachaparambil [43], *K*_1_ and *K*_2_ can be approximated as:

(32) $$K_{1}\left(\dot{\gamma }\right)\approx K_{a}{\dot{\gamma }}^{n}$$

(33) $$K_{2}\left(\dot{\gamma }\right)\approx K_{b}{\dot{\gamma }}^{m}$$

where *K_a_*, *K_b_*, *n*, and *m* are the calculating parameters. 

Subsequently, through mathematical derivation, we express the parameters in the foundational equation as solutions dependent on time and shear rate. The specific steps of the derivation are as follows:

It is necessary to connect function *f*_1_ with existing parameters, thus leading to the construction of an equation:

(34) $$f_{1}\left(\dot{\gamma }\right)=K_{l}{\dot{\gamma }}^{l}$$

For particle decomposition during stirring, i.e., $K_{1}\left(\dot{\gamma }\right)=0$, when coalescence is neglected, the intrinsic Equation (30) is built in a function form as:

(35) $$\tau \approx C_{01}+C_{11}{\dot{\gamma }}^{D_{1}}+\lambda_{t=t_{0}}K_{I}{\dot{\gamma }}^{l}e^{-K_{b}{\dot{\gamma }}^{m}\left(t-t_{0}\right)}$$

when coalescence is not neglected, $K_{2}\left(\dot{\gamma }\right)=0$, then we can get

(36) $$\tau \approx C_{02}+C_{12}{\dot{\gamma }}^{D_{2}}+K_{ll}{\dot{\gamma }}^{ll}+K_{ll}{\dot{\gamma }}^{ll}\left(\lambda_{t=t_{0}}-1\right)e^{-K_{a}{\dot{\gamma }}^{n}\left(t-t_{0}\right)}$$

To differentiate the parameters or functions in two distinct scenarios, subscripts have been added to *C*_0_, *C*_1_, *D*, and *K*, respectively. Furthermore, considering the role of hydration and nanoclay, in the decomposition phase, the parameters *C*_11_ and *C*_12_ have been transformed into an equation that relates to both via Equations (37) and (38):

(37) $$\tau \approx C_{01}+C_{11}\left(n_{\mathrm{NC}},Q_{\max}\frac{d\alpha_{d}}{dt}\right){\dot{\gamma }}^{D_{1}\left(n_{\mathrm{NC}},t_{0}\right)}+C_{11}\left(n_{NC},t_{0}\right){\dot{\gamma }}^{l}e^{-K_{b}{\dot{\gamma }}^{m}\left(t-t_{0}\right)}$$

In the coalescence phase, combining $K_{ll}$ with $\left(\lambda_{t=t_{0}}-1\right)$:

(38) $$\tau \approx C_{02}+C_{12}\left(n_{\mathrm{NC}},Q_{\max}\frac{d\alpha_{d}}{dt}\right){\dot{\gamma }}^{D_{2}}+K_{ll}{\dot{\gamma }}^{ll}+K_{l2}{\dot{\gamma }}^{ll}e^{-K_{a}{\dot{\gamma }}^{n}\left(t-t_{0}\right)}$$

Then by combining $C_{1k}\left(n_{\mathrm{NC}},Q_{\max}\frac{d\alpha_{d}}{dt}\right)$(*k* = 1, 2) and $D_{1}\left(n_{\mathrm{NC}},t_{0}\right)$, we can obtain a new function by taking the 1st order term approximation for various expansions:

(39) $$C_{1k}\left(n_{\mathrm{NC}},Q_{\max}\frac{d\alpha_{d}}{dt}\right)\approx G_{k0}+G_{k1}{n_{\mathrm{NC}}}^{\lambda_{k}}+H_{k1}n_{\mathrm{NC}}+A_{0k}+A_{1k}\left(Q_{\max}\frac{d\alpha_{d}}{dt}\right)+A_{2k}{\left(Q_{\max}\frac{d\alpha_{d}}{dt}\right)}^{2}+A_{3k}{\left(Q_{\max}\frac{d\alpha_{d}}{dt}\right)}^{0.5}$$

(40) $$D_{1}\left(n_{\mathrm{NC}},t_{0}\right)=D_{10}+t_{0}\frac{D_{20}+D_{30}{n_{\mathrm{NC}}}^{D_{40}}}{t_{0\max}}$$

where $t_{0\max}$ is the maximum settling time in each settling sample after mixing. In the coalescence stage, for further simplification, $K_{l2}$ is considered to have the same expression with $C_{12}\left(n_{\mathrm{NC}},Q_{\max}\frac{d\alpha_{d}}{dt}\right)$. Then the intrinsic model of the whole hysteresis loop process is:

(41) $$\tau \approx \left\{\begin{array}{cc} C_{01} & +C_{11}\left(n_{\mathrm{NC}},Q_{\max}\frac{d\alpha_{d}}{dt}\right){\dot{\gamma }}^{D_{1}\left(n_{\mathrm{NC}},t_{0}\right)}+C_{11}\left(n_{\mathrm{NC}},t_{0}\right){\dot{\gamma }}^{l}e^{-K_{b}{\dot{\gamma }}^{m}\left(t-t_{0}\right)},Breakdown\\ & C_{02}+C_{12}\left(n_{\mathrm{NC}},Q_{\max}\frac{d\alpha_{d}}{dt}\right){\dot{\gamma }}^{D_{2}}+K_{ll}{\dot{\gamma }}^{ll}+K_{l2}{\dot{\gamma }}^{ll}e^{-K_{a}{\dot{\gamma }}^{n}\left(t-t_{0}\right)},\mathrm{Build-up} \end{array}\right.$$

This is the intrinsic structure relationship of the slurry of cementitious material considering simultaneously the settling time, hydration, nanoclay, shear rate, and thixotropic hysteresis effect.

## 3. Results and Discussion

### 3.1. The First Exothermic Peak—Calibration and Validation 1

Based on the results of Teng et al. [44] for ultra high performance concrete (UHPC), using the results without Ghanami clay, the first group of calculating parameters in Equation (23) could be derived through calibration from the value of NC-0 [44] as:

(42) $$V_{0}=-86.04944,\;V_{1}=93.2238,\;b=\;0.082726,\;\eta =-1.001$$

The second group of calculating parameters in Equation (23) were calibrated with the exothermic curve with external nanoclay doped with 0.25%:

(43) $$W_{1}=-0.137132,\;\beta_{1}=2.001,\;U_{1}=1.1022,\;\zeta =1.0001,\;\xi =1$$

**Figure 1:**
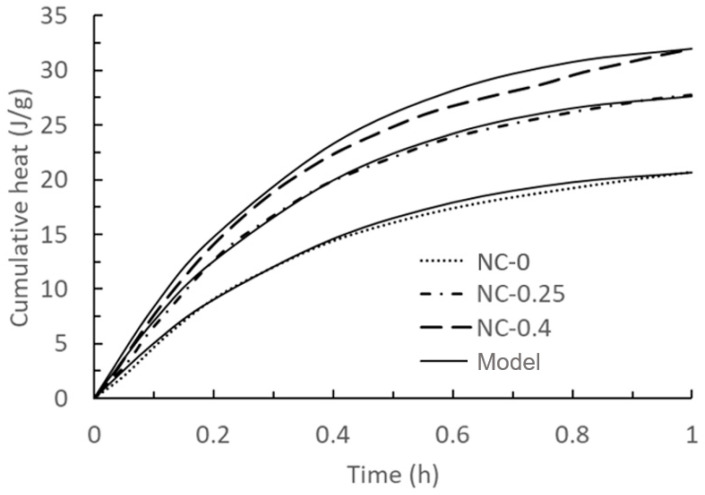
Comparison of model results of the first exothermic peak of hydration of cement slurry mixed with 0%, 0.25%, and 0.4% nanoclay and experimental data taken from the literature [44]. Solid lines: model results with NC-0 and NC-0.25 used for calibration, and NC-0.4 as a validation. Dashed lines: experimental data.

After inserting all the calculating parameters in Equation (23) and plotting cumulative heat over time, the comparison with the exothermic curve of 0.4% with Teng’s work is shown in Figure 1. The calculating parameters remain unchanged during the plotting process.

It is noticeable that initially, the cumulative heat rises rapidly, followed by a gradual slowdown in the rate of increase. This phenomenon can be ascribed to the formation of hydration products covering the surface of binder particles, which delays hydration [45]. Moreover, the cumulative heat of UHPC mortar within the first hour was augmented with an increasing NC content in the mixtures.

### 3.2. The First Exothermic Peak—Calibration and Validation 2

Using the results of Quanji et al. [14], the calculating parameters of the first exothermic peak heat flow curve of the cement slurry with unadulterated clay were calibrated as:

(44) $$V_{0}=19.061372,\;V_{1}=12.25363,\;b=\;4.664751,\;\eta =-3.2001$$

Based on this, the nanoclay content of 3% was chosen as the benchmark to calibrate the other parameters as:

(45) $$W_{1}=0.8724666,\;\beta_{1}=-10.72375,\;U_{1}=0.38694726,\;\zeta =0.5001,\;\xi =1$$

**Figure 2:**
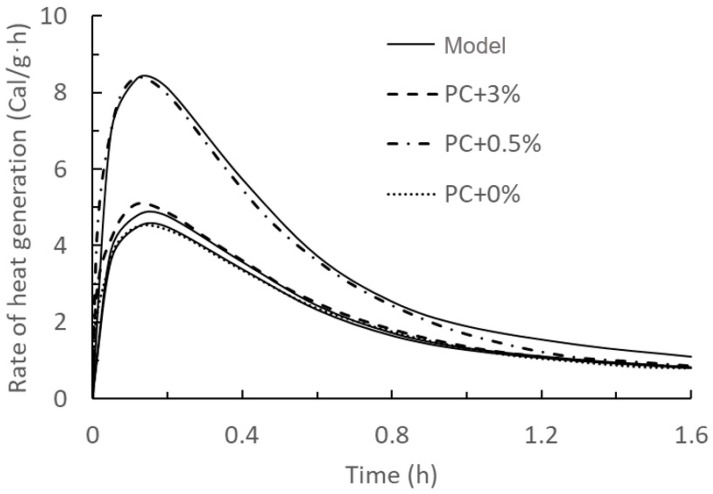
Comparison of model results of the first exothermic peak of hydration of Portland cement (PC) slurry mixed with 0%, 0.5%, and 3% nanoclay and experimental data taken from the literature [14]. Solid lines: model results with PC + 0% and PC + 3% used for calibration, and PC + 0.5% as a validation. Dashed lines: experimental data.

**Figure 3:**
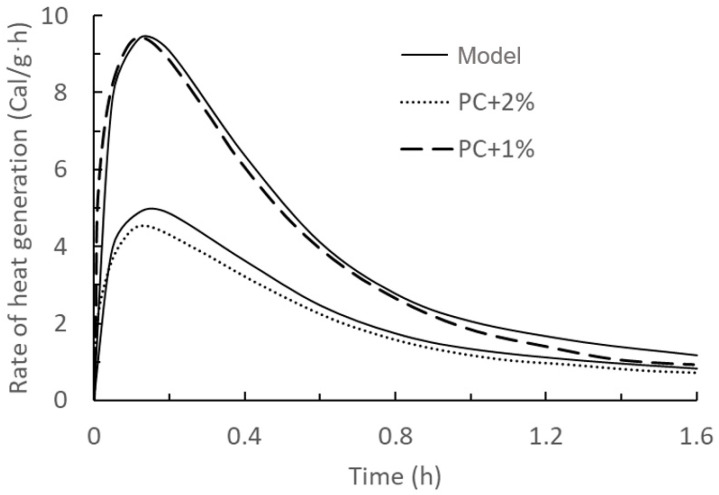
Comparison of model predictions of the first exothermic peak of hydration of cement slurry mixed with 1% and 2% nanoclay and experimental data (dashed line) taken from the literature [14]. Solid lines: model results with PC + 1% and PC + 2% as validations. Dashed lines: experimental data.

After inserting all the calculating parameters in Equation (23) and plotting rate of heat generation over time, the results were compared with Quanji’s work as shown in Figure 2 and Figure 3. 

From the comparison between the current model with the results from Teng and Quanji’s work, the predicted results of this first exothermal peak model are in good agreement with the measured results, which indicates that the accuracy of this model is relatively high. Additionally, the type and source of nanoclay have no impact on the accuracy of the model.

### 3.3. The Rheological Behavior—Calibration and Validation 3

In model validation 3, we will mainly compare the effect of shear rate on hysteresis. Combining with the measured results of Quanji et al. [14], the hysteresis curve model is calculated by calibrating the parameters using the measured results of Portland cement (PC) + 0% NC, PC + 0.5% NC at 0 min and PC + 0.5% NC at 75 min; with microstructural break-down as Equation (46) and microstructural build-up as Equation (47):

(46) $$\begin{array}{l} \tau \approx {\dot{\gamma }}^{0.01+t_{0}\frac{0.22-0.03{n_{\mathrm{NC}}}^{1.183}}{t_{0\max}}}\{35+14{n_{\mathrm{NC}}}^{1.504}\\ \;\;\;\;\;\;+\left[25,366.747+38,824.958\left(Q_{\max}\frac{d\alpha_{d}}{dt}\right)-4899.634{\left(Q_{\max}\frac{d\alpha_{d}}{dt}\right)}^{2}-59,193.898{\left(Q_{\max}\frac{d\alpha_{d}}{dt}\right)}^{0.5}\right]\}(17.5\\ \;\;\;\;\;\;+7{n_{\mathrm{NC}}}^{1.503}+t_{0}\frac{78.5+181.279{n_{NC}}^{0.958}-85.179n_{NC}}{t_{0\max}}){\dot{\gamma }}^{0.05}e^{-\frac{t-t_{0}}{2}} \end{array}$$

(47) $$\begin{array}{l} \tau \approx \{20+7{n_{\mathrm{NC}}}^{1.129}-2.401n_{\mathrm{NC}}\\ \;\;\;\;\;\;+[8153.894+12,538.771\left(Q_{\max}\frac{d\alpha_{d}}{dt}\right)-1586.163{\left(Q_{\max}\frac{d\alpha_{d}}{dt}\right)}^{2}\\ \;\;\;\;\;\;-19,097.797{\left(Q_{\max}\frac{d\alpha_{d}}{dt}\right)}^{0.5}]\}\left({\dot{\gamma }}^{0.35}+{\dot{\gamma }}^{0.05}e^{-\frac{t-t_{0}}{2}}\right) \end{array}$$

**Figure 4:**
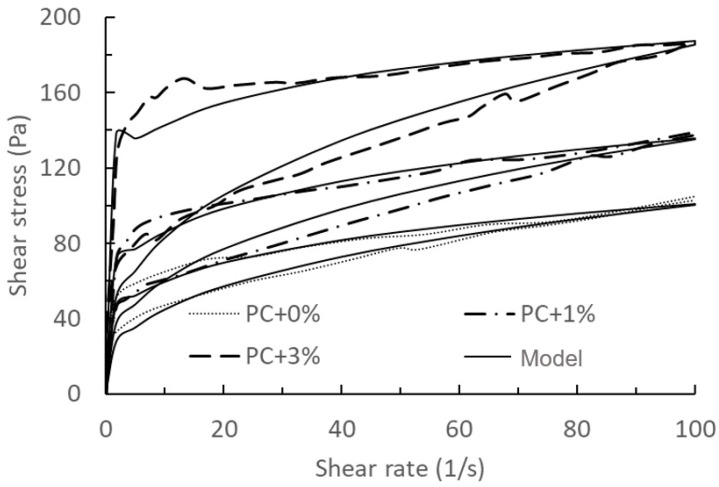
Comparison of model results of the thixotropy behaviour with 0%, 1%, and 3% nanoclay and experimental data at *t*_0_ = 0 min taken from the literature [14]. Solid lines: model results with PC + 0% used for calibration, and PC + 0.1% and PC + 3% as validations. Dashed lines: experimental data.

**Figure 5:**
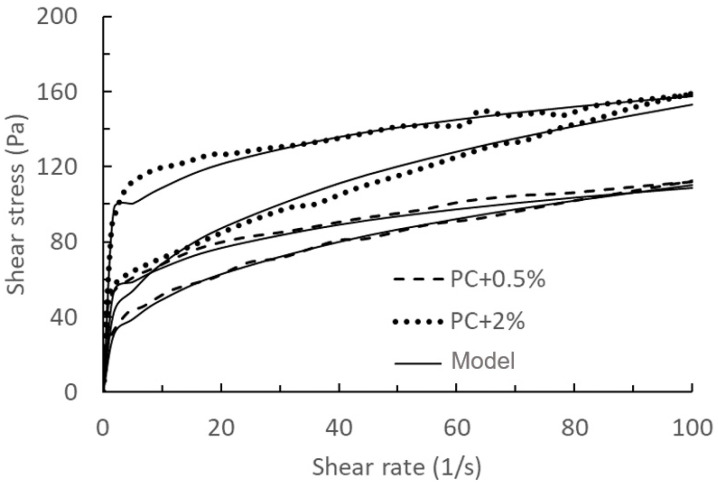
Comparison of model results of the thixotropy behaviour with 0.5% and 2% nanoclay and experimental data at *t*_0_ = 0 min taken from the literature [14]. Solid lines: model results with PC + 0.5% used for calibration, and PC + 2% as a validation. Dashed lines: experimental data.

**Figure 6:**
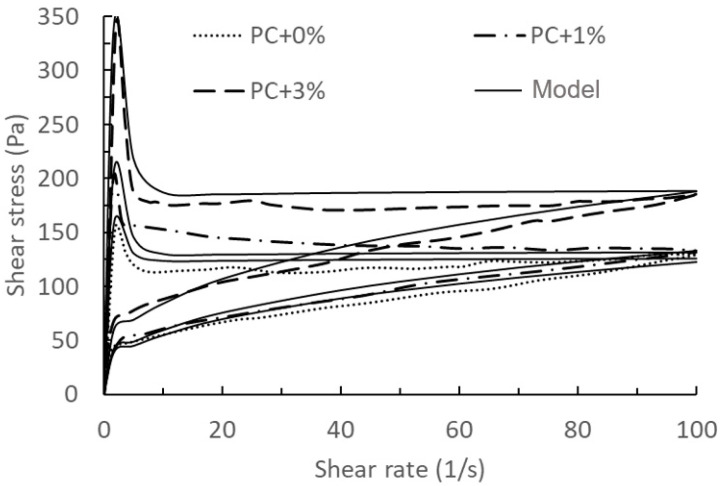
Comparison of model predictions of the thixotropy behaviour with 0%, 1%, and 3% nanoclay and experimental data at *t*_0_ = 75 min taken from the literature [14]. Solid lines: model results with PC + 0%, PC + 1% and PC + 3% used for validations. Dashed lines: experimental data.

**Figure 7:**
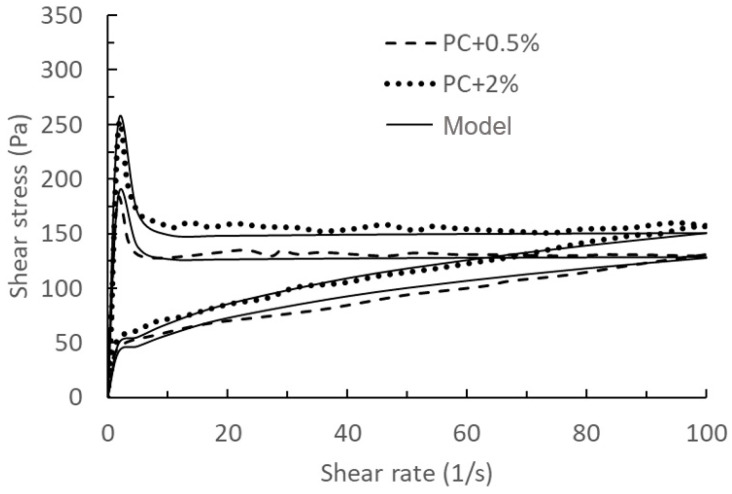
Comparison of model results of the thixotropy behaviour with 0.5% and 2% nanoclay and experimental data at *t*_0_ = 75 min taken from the literature [14]. Solid lines: model results with PC + 0.5% used for calibration, and PC + 2% as a validation. Dashed lines: experimental data.

Based on different hydration times (0 min and 75 min), the model’s validation and prediction are divided into two groups, as shown in Figure 4, Figure 5, Figure 6 and Figure 7.

Combining with the conclusions drawn by Quanji [14], the disparity in thixotropy in all cement pastes between the upward and downward curves for pastes with different nanoclay amounts notably widens, indicating an increase in thixotropy. Nanoclay addition also heightened the rate of cement paste hydration, showing a strong correlation between heat generation rate and structural rebuilding. Different nanoclay dosages exhibited varying rates of heat generation and structural rebuilding, with higher heat generation correlating with faster structural rebuilding and vice versa.

Comparing the results of Figure 4, Figure 5, Figure 6 and Figure 7, we can find that the measured results of Figure 6 and Figure 7 for this model have a better agreement, while there is some deviation from the results of Figure 4 and Figure 5, especially at the beginning of the breakdown process. The possible reason for this deviation is as follows: in the actual experimental test, it is not possible to ensure that the cement does not continue to hydrate completely during the test, especially for samples with a measurement time of 0 min. The deviation between the mathematical model and the experimental results could be seen as a systematic error in the actual experimental test, which could be justified or calibrated by the suitable choice of the mathematical model.

### 3.4. The Rheological Behavior—Calibration and Validation 4

In model validation 4, we will mainly compare the effect of cement hydration time on hysteresis. Based on the empirical results of Kawashima et al. [46], for a given degree of hydration, it is considered that the degree of hydration does not change much throughout the test, and the calculating parameters of the decomposition phase of the above model are calibrated using a shear rate of 50 s^−1^ with 0% nanoclay content, and a shear rate of 300 s^−1^ with 0% nanoclay content. When the degree of hydration is a fixed value, then Equation (41) can be simplified as:

(48) $$\tau =169.730+\left(-576.290+2410.377n_{\mathrm{NC}}\right){\dot{\gamma }}^{-0.447}+\left(0.899-0.0519{n_{\mathrm{NC}}}^{0.5}\right){\dot{\gamma }}^{0.625}e^{-0.16t}$$

**Figure 8:**
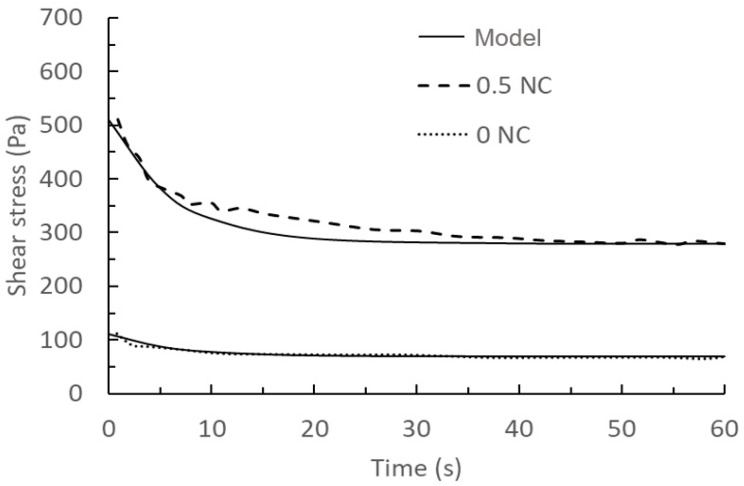
Comparison of model results of the rheology behaviour with 0% and 0.5% nanoclay and experimental data at a shear rate of 50 s^−1^ taken from the literature [46]. Solid lines: model results with 0NC used for calibration, and 0.5NC as a validation. Dashed lines: experimental data.

**Figure 9:**
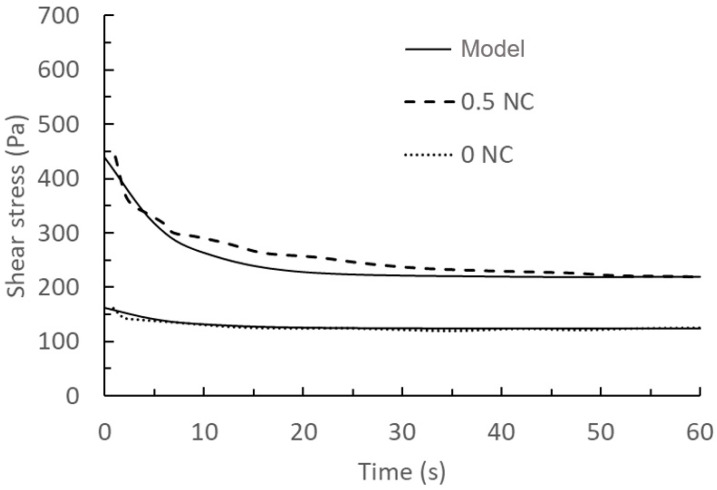
Comparison of model results of the rheology behaviour with 0% and 0.5% nanoclay and experimental data at a shear rate of 300 s^−1^ taken from the literature [46]. Solid lines: model results with 0NC used for calibration, and 0.5NC as a validation. Dashed lines: experimental data.

Comparison of the above prediction results and the measured results at a shear rate of 300 s^−1^ with a nanoclay content of 0.5% are shown in Figure 8 and Figure 9.

Table 1 reflects the deviation between the predicted results of our model and the experimental data, using the Root Mean Squared Error (RMSE) method. Overall, the model can accurately (overall below 3% error) predict the rheological properties of cement after the addition of nanoclay. Also, the results of the two comparisons produced relatively large deviations between the model and the experimental test results at the earlier hydration time stage, while the consistency increased gradually with increasing hydration time until the deviations completely disappeared. This also confirms the analysis in model validation 3 that the relative systematic errors caused by the time interval in the actual experimental tests, perhaps due to instrument scanning and human operation gradually disappearing as the hydration time and degree of cement hydration increase.

## 4. Conclusions

This article simplifies the early reactions of cement with added nanoclay into a dissolution/nucleation process. A simplified empirical mathematical model to predict the first exothermic peak in the hydration process is derived from the Avrami and Cahn equations and the rheological characteristics based on the work of Cheng and Evans. 

The first exothermic peak model is defined by two sets of calculation parameters. The equation describing the rate of exothermic reaction over time follows an exponential pattern, enabling the anticipation of alterations in both cumulative heat and heat rate during hydration time, accounting for different concentrations of nanocaly. In the rheological model, the connection between shear stress, shear rate, and time is portrayed by an exponential product relationship. It allows for the prediction of changes in shear stress with one variable while keeping the other variable constant, which could be time or shear rate. Upon comparison with existing experimental data and empirical models, this model closely aligns with experimental data for systems hydrated for about an hour, and shows greater deviation in systems with the very initial hydration period. This discrepancy is likely due to the challenge of completely avoiding cement hydration during experimental measurements. The proposed analytical model couples cement hydration and rheology mechanisms in a simplified way to enable a relatively easy use for studying sensitivity of influencing parameters.

The model proves accurate, reliable, and applicable for quantitatively characterizing and assessing the rheological characteristics of nanoclay-modified cementitious materials in early fresh state. This serves as a valuable tool for researchers and engineers to enhance the understanding and control of the early hydration process and fresh rheology characteristics in cementitious materials incorporating nanoclay, leading to advancements in concrete printing techniques and design strategies.

## Figures and Tables

**Table 1:** Root Mean Squared Error between simulated data and experimental data.

Figure	Name	RMSE	Figure	Name	RMSE
Figure 1	NC-0	0.13 J/g	Figure 5	PC + 0.5%	2.92 Pa
	NC-0.25	0.05 J/g		PC + 2%	9.07 Pa
	NC-0.4	0.35 J/g	Figure 6	PC + 0%	7.52 Pa
Figure 2	PC + 0%	0.11 Cal/g·h		PC + 1%	1.88 Pa
	PC + 0.5%	0.18 Cal/g·h		PC + 3%	12.90 Pa
	PC + 3%	0.26 Cal/g·h	Figure 7	PC + 0.5%	8.37 Pa
Figure 3	PC + 1%	0.21 Cal/g·h		PC + 2%	9.92 Pa
	PC + 2%	0.34 Cal/g·h	Figure 8	0 NC	4.12 Pa
Figure 4	PC + 0%	3.68 Pa		0.5 NC	25.69 Pa
	PC + 1%	6.78 Pa	Figure 9	0 NC	3.32 Pa
	PC + 3%	11.27 Pa		0.5 NC	21.45 Pa

## Data Availability

The data are available upon request from the corresponding author.
